# Predicting Advanced Prostate Cancer from Modeling Early Indications in Biopsy and Prostatectomy Samples via Transductive Semi-Supervised Survival Analysis

**DOI:** 10.1155/2018/2178645

**Published:** 2018-10-21

**Authors:** Faisal M. Khan

**Affiliations:** Rutgers, The State University of New Jersey, Piscataway, NJ 05584, USA

## Abstract

Prostate cancer is the most prevalent form of cancer and the second most common cause of cancer deaths among men in the United States. Accurate prognosis is important as it is the principal factor in determining the treatment plan. Prostate cancer is a complex disease which advances in stages. While clinical failure (including metastasis) is a significant endpoint following a radical prostatectomy, it can often take years to manifest, usually too late to be optimistically treated. In practice, the earlier endpoint of PSA Recurrence is frequently used as a surrogate in prognostic modeling. The central issue in these models is managing censored observations which challenge traditional regression techniques. The true target times of a majority of instances are unknown; what is known is a censored target representing some earlier indeterminate time. In this work we apply a novel transduction approach for semi-supervised survival analysis which has previously been shown to be powerful in medical prognosis. The approach considers censored samples as semi-supervised regression targets leveraging the partial nature of unsupervised information. We explore the use of this approach in building prostate cancer progression models from multimodal characteristics extracted from both biopsy and prostatectomy tissues samples. In this work, the approach leads to a significant increase in performance for predicting advanced prostate cancer from earlier endpoints and may also be useful in other diseases for predicting advanced endpoints from earlier stages of the disease.

## 1. Introduction

Prostate cancer is the most prevalent form of cancer and the second most common cause of cancer morbidity among men in the United States. The most common treatment is the surgical removal of the prostate through a radical prostatectomy (RP). Unfortunately, RP is no guarantee of a cure. Approximately 3-5% of men after RP experience significant clinical failure (CF) including metastasis and/or death-of-cancer. While CF is a clinically meaningful endpoint, it can often take years to present; and when it does the disease may be too advanced for effective treatment. Therefore, an earlier endpoint of prostate-specific-antigen-recurrence (PSAR) after RP is frequently employed as a surrogate. This is however a noisier endpoint, which 15-25% of men experience after RP. Not everyone with PSAR progresses to the more advanced stage of CF. Since PSAR occurs years earlier though, a physician and patient can start to make complex decisions about treatment options and impact on quality of life. Accurate prognosis is important as it is the principal factor in determining the treatment plan. In prognostic modeling, PSAR data is frequently employed to predict CF [1–3].

While such time-to-event prediction can pose a regression problem, survival analysis is challenging since data in such circumstances is characterized by censored observations. The term “censoring” in biostatistics describes the fact that the target survival time is not known for all samples. For instance, patients might not experience death or cancer relapse during the course of a study or be lost to follow-up. The only time known is their last record of being healthy; hence the regression target time is uncertain and only known “up-to-a-point.” This is distinctly different from the notion of missing data [4–6].

Censored observations contribute incomplete information since the event of interest may occur after patients are lost to follow-up. Omitting the censored samples [7] and treating them as nonrecurring samples in a classifier [8] both bias the resulting model and should be avoided. Additionally, in healthcare diagnostics, due to the costs of identifying acceptable patients who will provide consent for inclusion in research, and then actively tracking them over a significant period of time, the sample size is often small, in the tens or hundreds. Since most of the samples may be censored (91% in prostate cancer [2]) dropping such patients is a very unattractive option and accounting for them is of crucial importance. Survival analysis represents a special example of the typical complexity in modeling noisy high-dimensional biomedical data to predict complex medical phenomena.

There has been extensive research into algorithms and techniques for survival analysis [5, 6, 9, 10]. A recent innovation has been to consider censored samples as semi-supervised targets. While there has been significant work in semi-supervised classification [11–13], there has been limited work in semi-supervised regression [14]. Work has treated samples as either fully labeled or unlabeled and did not take into account the partial nature of unsupervised information, as is the case in time-to-event medical prognosis problems. We have recently proposed a novel framework which modifies survival analysis algorithms by transducing appropriate target times in a semi-supervised regression context [4, 15]. This framework has already been employed to model prostate cancer with multimodal imaging and clinical characteristics extracted from both biopsy and prostatectomy samples [16, 17].

Until recently the approach has only been applied to directly predict a medical prognostic endpoint. In the present paper, we consider the interesting and practical problem where an earlier disease endpoint is used to predict a later one. We concentrate on the highly relevant prostate cancer space as, unlike other cancers, prostate cancer has a long multiyear horizon with multiple stages of the disease

## 2. Overview of Survival Analysis and Semi-Supervised Regression

### 2.1. Overview of Survival Analysis

Healthcare data for prognostic modeling is usually obtained by tracking patients over the course of time in a well-designed study, perhaps lasting years. Often a predefined event such as the relapse of a disease or death due to disease is the focus of the study. The major difference between survival analysis and other regression problems is that the event of interest is frequently not observed in many of the subjects. Patients who did not experience the endpoint during the study or were lost to follow-up for any cause (i.e., the patient moved during a multiyear study) are considered as* censored*. All that is known about them is that they were disease-free up to a certain point, but what occurred subsequently is unknown. They may have actually experienced the endpoint of interest at a later time. Conversely, patients who have experienced the endpoint of interest are considered as* noncensored* samples or* events*. In many medical prognosis problems, the vast majority of instances (as high as 96%) can be censored. The incomplete nature of the outcome thus challenges traditional regression techniques. Methods which can correctly account for censored observations are essential [6, 9, 10, 15].

If *T*_*i*_ denotes the actual target time, *C*_*i*_ the censored time, and *U*_*i*_ the observed time for all patients, then for measured events *U*_*i*_ = *T*_*i*_ and for censored cases *U*_*i*_ = *C*_*i*_ < *T*_*i*_. The survival outcomes for* n* patients are then represented by pairs of the random variables (*U*_*i*_, *δ*_i_) for* i* = 1,…,* n*. *δ*_i_ indicates whether the observed survival time *U*_*i*_ corresponds to an event (*δ*_i_ = 1) or is censored (*δ*_i_ = 0). Given a* d-*dimensional vector *x*_*i*_  *Є*  **R**^d^, the data* D *for medical prognosis can be represented as

(1) D=Ui,xi,δini=1.

(2) Ui=min⁡Ti,Ci

(3) δi=ITi≤Ci=0,for  censored  observation,1,for  exact  observation.

### 2.2. Methods for Survival Analysis

The field of prognostic survival analysis has been primarily of interest to biostatisticians. The Cox proportional hazards model is the de facto standard approach [9, 10]; it estimates the log hazard for a patient as a linear combination of the patient's features, plus a baseline hazard. A patient's individual predicted hazard function predicts their survival time. A hazard function is the instantaneous rate of decline in survival at a point in time. The Cox model makes the assumption that the hazard functions for any two individuals are proportional; their ratio is constant over time. This assumption is reflected in the formula for the approach:

(4) $$\begin{array}{c} h_{i}\left(\;t\;\right)=\exp\left(\;\overset{p}{\underset{j=1}{\sum }}b_{j}X_{ij}\;\right)h_{0}\left(\;t\;\right) \end{array}$$

where *h*_*i*_*(t)* is the hazard function for the i^th^ individual, *b*_*j*_ is the slope term for the j^th^ feature, *X*_*ij*_ is the value of feature* j* for individual* i*, exp*()* refers to the exponential function (exp*(u) = e*^u^), and* h*_0_*(t)* refers to a hazard function for an individual with zeros for all features. Regression parameter estimates (the* b* terms) are obtained via maximum likelihood estimation. The Cox model only employs censored patients' data in calculating the hazard function up to the time of censoring; afterwards they are excluded.

Widespread adoption of Support Vector Machines (SVMs) has also led to recent applications for survival analysis [5, 6, 15]. One example is SVRc which adapts normal support vector regression through an asymmetric loss-penalty function depending on whether a patient's observation is censored or an event [5]:

(5) minW,b 12W2+∑i=1nCiξi+Ci∗ξi∗

given the constraints

(6) yi−W•Φxi+b≤εi+ξiW•Φxi+b−yi≤εi∗+ξi∗ξi∗≥0,  i=1…n

where

(7) si=1if  censored,si=0if  eventCi∗=siCc∗+1−siCn∗εi∗=siεc∗+1−siεn∗

### 2.3. Overview of Semi-Supervised Regression

The basic idea of transductive regression [14] is that, given* m* labeled data and labels (x_1_, y_1_),…, (x_m_, y_m_) as well as* u* unlabeled data points x_m+1_,…, x_m+u_, transductive regression learning algorithms must accurately predict the labels y_m+1_,…, y_m+u_. Reference [14] describes two basic steps for such algorithms. The first is local estimation where labels of unlabeled points are assigned based on their neighbors. In the second step, through optimization, a hypothesis is selected that best fits the known supervised labels and the estimated labels. While these approaches work well for problems with fully labeled and unlabeled instances, their direct adoption for survival analysis is not ideal as they do not leverage the partial information of true outcome in the censored times. Additionally, classical semi-supervised regression does not reflect the circumstances of survival analysis where more than 90% of the instances may be unsupervised but contain partial information. The scarcity of neighboring events with known target labels for censored instances challenges them.

To the best of our knowledge, leveraging the partial knowledge of true known outcomes in the encoded censored times for survival analysis is a largely neglected area. One of the first efforts [4, 15] developed a framework for transducing the appropriate censored times in a medical survival analysis problem. This framework is what we explore in this work

## 3. Materials and Methods

In this paper, we leverage the use of a transduction approach for semi-supervised regression in survival analysis to build prostate cancer models for PSAR which are used to then predict the later, more clinically meaningful endpoint of CF. Prostate cancer characteristics representing multiple modalities including clinical characteristics, quantitative protein biomarker expression, and microscopic image analysis are employed.

### 3.1. Semi-Supervised Regression through Transduction

As discussed, the ability to leverage the incomplete information in the censored samples of time-to-event problems could provide significant advantages. If the “true” target as opposed to the censored target was known, the performance of predictive models would be improved.

Reference [15] presents an innovative approach that is, in essence, a wrapper around any regression function, whether developed for survival analysis or not. For each censored case (*U*_*i*_, *δ*_i_ = 0), it iterates through possible target values between *U*_*i*_ and* T*_max_ (the maximum observed time* U* in the dataset). It then transduces or chooses a new target time $\hat{U}T_{i}$ which improves accuracy, maximizing some criterion for measuring predictive performance. The approach is extremely flexible, able to work with almost any regression function* F()* and measure of accuracy* Criterion (y, t)*. Given a dataset *D* = {*U*_*i*_, *x*_*i*_, *δ*_*i*_}^*N*^_*i*=1_, the algorithm can be described as

(8) maxCriteriony,U,U^T y=FD=Ui,xi,δii=1n

given the constraints

(9) Tmax=max⁡Ui=1,…,nUi≤U^Ti≤Tmax;if  δi=0U^Ti=Ui;if  δi=1

A key issue is how to explore the space of possible target values in an efficient manner. Semi-supervised classification algorithms initially employed an exhaustive method assigning each class label to every unlabeled instance, in order to transduce the optimal label. Unfortunately, this led to a transduction complexity of C^n^ where C is the number of classes and* n* the number of unlabeled instances. Accordingly, researchers began to develop computationally more reasonable methods.

Our semi-supervised regression approach exploits a censored instance's own partial information of true outcome rather than its neighbor's labels to transduce optimal target times. The censored time represents the minimum possible value of the true target. The optimal target for each censored instance could thus be transduced by testing values in increments from the censored time. The initial idea was to replicate the exhaustive search of semi-supervised classification [11, 12], but this is impractical. In one sample dataset, an average of 10 target values per each of the 340 censored cases would result in a transduction complexity of 10^340^.

From a theoretical computer science perspective, the algorithm would have logarithmic complexity of O (10^n^) [18, 19]. To avoid this, the proposed technique is a singular transduction procedure which avoids the exhaustive method. Each instance is treated independently, and the best time for each censored case is found independent of the other censored cases. This results in a slight modification to (8) for a singular rather than exhaustive transduction approach with linear complexity O (10n):

(10) $$\begin{array}{c} {\left(\;\underset{Criterion\left(y,U\right),\hat{U}T_{i}}{max}\;y=F\left(\;D={\left\{\;U_{i},x_{i},\delta_{i}\;\right\}}_{i=1}^{n}\;\right)\;\right)}_{i=1}^{n}. \end{array}$$

One subtle but crucial point to note is that when evaluating the fit of the model on the training data, the evaluation should be done with the original censored times rather than the new transduced times. Otherwise the resulting performance metrics may artificially be inflated as they will be calculated on the discovered targets that were derived precisely to improve performance. Subsequently when testing on an independent validation set where it is not possible to transduce the times but to simply apply the model, the resulting model would grossly overfit, as was observed by the authors. This is exactly why in (8) and (10) we maximize* Criterion (y, U) *with respect to known data rather than* Criterion (y, *$\hat{U}T$*).*

In this paper, we employ the framework for the application of exploring subsequent disease progression from earlier indications, a heretofore unexplored area of research. We empirically study two applications of this framework which have previously proven successful, for the Cox model and SVRc [4, 15]

### 3.2. Performance Metrics

In conventional regression, a useful accuracy metric is the error in predicting the targets. However, in survival analysis this is not possible due to censoring. For events, the prediction error can be assessed. For censored records, predictions are wrong only if less than the targets; otherwise the error, if any, is unknown. This requires different performance criteria.

The concordance index (CI) is the standard metric used for assessing the predictive ability of a survival model [5, 9]. The CI measures the concordance between model results and the survival times. Survival analysis is inherently a ranking problem and the CI measures the accuracy of ranking a model's results against the actual survival times. It is the probability that a patient with a shorter survival time will have a smaller predicted value. It ranges from 0 to 1, with 0.5 indicating an absence of correlation, a random result. A value of 0 indicates perfect negative correlation, and 1 perfect positive correlation. The CI is a linear transform of Somers' d statistic and is similar in interpretation to the area-under-the-curve and the Mann–Whitney statistics [20].

A survival model in medicine often helps stratify a patient population into high and low risk groups. Diverse risk profiles can lead to better targeted therapies and disease management. For a specific time point, patients can be stratified into high and low risk groups based on a model's predictions. The positive class identifies patients who were events prior to this time point and the negative class of patients (events or censored) with targets occurring after this time point. Censored patients with targets prior to the time point are excluded. Thus, in addition to the CI, the ability to correctly identify high and low risk groups is measured via sensitivity and specificity. Since censored patients earlier than the time point are excluded, it is often a good idea to look at the CI and the classification metrics at the same time.

Both the CI and the sensitivity-specificity pairing are metrics independently used in the medical literature [1–3]. We employed a criterion to simultaneously assess the CI and the product of the sensitivity and specificity. The product of the sensitivity and specificity is a good measure that has the same scale of accuracy as the CI. While, in absolute theory, the CI may not have the same range as the product of sensitivity and specificity because CI values less than 0.5 imply negative correlation (similar to the AUC), since all useful models must have CIs greater than 0.5, this is not problematic from a practical perspective. Consequently, in all the presented experiments the performance criterion for evaluation was

(11) $$\begin{array}{c} \text{Criterion}=\text{CI}+\left(\;\text{Sensitivity}*\text{Specificity}\;\right) \end{array}$$

### 3.3. Prostate Image Analysis

For patients with prostate cancer, clinicians aim to develop an individualized treatment plan based on a mechanistic understanding of the disease factors unique to each patient. Key characteristics include clinical measurements such as the level of PSA (prostate specific antigen) and the Gleason Grade [21, 22]. Additional characteristics can be extracted from other modalities such as algorithmic analysis of various types of prostate images. Two main information sources are the architecture of the tumor morphology and biomolecular mechanisms of the disease as assessed by biomarkers [3, 23–26]. There has been significant research in image analysis of prostate morphology as well as automated quantification of molecular and protein biomarker expression [24–26]. These quantitative image analyses from multiple modalities have become prevalent, yielding not only independent prognostic predictors of outcome but also features which can be combined into multivariate models [3]. In this work, we explore morphometric features from H&E (Hematoxylin and Eosin) and IF (immunofluorescent) images, as well as IF biomarker features [16].

### 3.4. H&E (Hematoxylin and Eosin) Metrics

Morphological and architectural characteristics of the prostate tissue, such as epithelial nuclei and cytoplasm, provide critical information for the diagnosis, prognosis, and therapeutic decision-making of prostate cancer. The subjective and highly variable Gleason grade assessed by expert pathologists from Hematoxylin and Eosin (H&E) stained specimens has been the standard for prostate cancer diagnosis and prognosis.

There has been significant work in automatically approximating the Gleason grade and quantifying other aspects of prostate morphology [24, 27–29]. The majority of proposed approaches consider various tissue components such as lumens, nuclei, and cytoplasm independently. The entire glandular unit of epithelial nuclei, cytoplasm, and stroma around a lumen provides a more accurate and comprehensive morphological assessment of disease severity.

**Figure 1:**
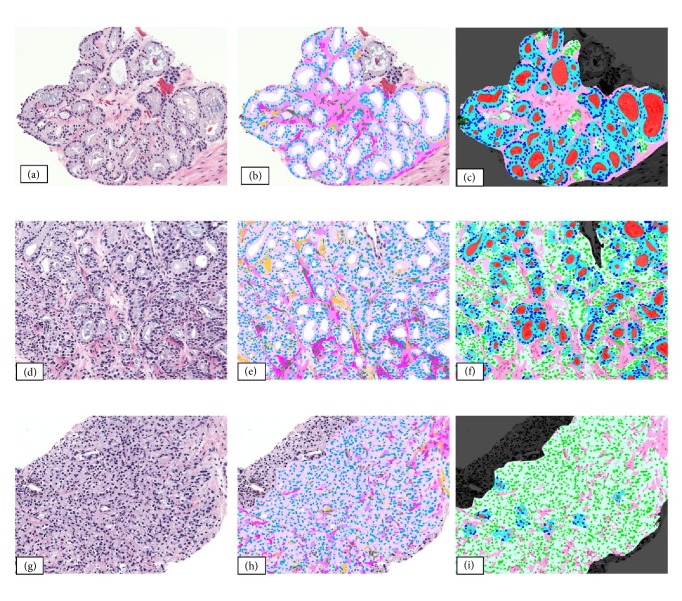
Images representing prostate cancer grades 3 (a-c), 4 (d-f), and 5 (g-i). Images representing the original H&E stain (a, d, g), primary object segmentation (b, e, h), and glandular object classification (c, f, i) are presented [24].

Methods we leveraged include one proposed by Fogarasi et al. [24] for automated analysis of gland unit features from H&E images. The approach initially segments and classifies primary cellular components such as cytoplasm, nuclei, stromal fibroblasts, lumens, blood vessels, and artifacts. This segmentation relies on cellular properties such as distance of tumor cells from lumens, as well as color, shape, texture, and neighborhood properties. The relationships between these components are analyzed and leveraged to construct distinct “gland units.” Biological characteristics such as logical and relative object positioning are employed to develop initial seeds which are optimized in an iterative classification process [17].  Figure 1 illustrated these gland units in segmented H&E images.

### 3.5. Immunofluorescence Morphology and Biomarkers

**Figure 2:**
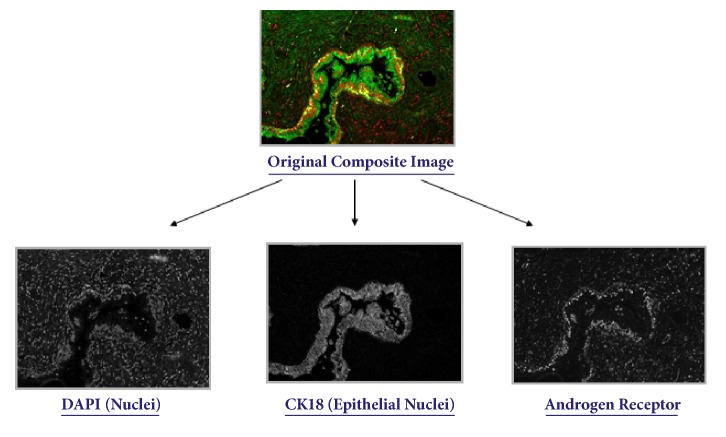
Sample composite image of a prostate gland spectrally unmixed into individual images representing DAPI, CK18, and AR biomarkers [17].

In multispectral immunofluorescence (IF) microscopy [25, 26, 30], multiple proteins in the tissue specimen are simultaneously labeled with different fluorescent dyes. Each dye has a distinct emission spectrum and its associated antibody binds to its target protein within a tissue compartment (i.e., nuclei or cytoplasm). The stained slide is illuminated under a fluorescence microscope with a light source for a specific wavelength. This excitation light is absorbed by the fluorescent dye causing it to emit light of a longer wavelength. The intensity of the emitted light is a measure of the target protein's concentration. In multiplexed IF images, the tissue is labeled with several antibodies at the same time. Each antibody is labeled with a unique fluorescent dye with distinct spectral characteristics. The tissue is then imaged with a multispectral camera and then spectrally unmixed, to yield multiple images with one image per individual dye/antibody. Two common dyes that reveal the tissue structure are DAPI (a nuclear stain) and CK18 (which stains epithelial cytoplasm). Nuclear objects are segmented and then separated using a colocalization scheme into epithelial nuclei positive for both DAPI and CK18 and stromal nuclei positive for DAPI but not CK18. Subsequently prognostic biomarkers such as AR (androgen receptor) are evaluated within each colocalized compartment.  Figure 2 illustrates a sample prostate gland unmixed into DAPI, CK18, and AR specific images [16, 17].

**Figure 3:**
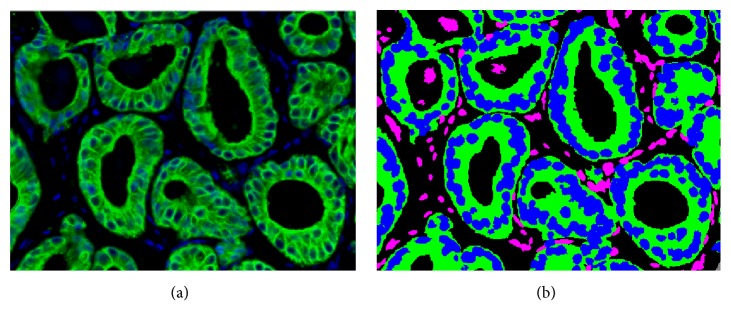
A multiplex IF pseudocolor image consisting of the DAPI counterstain (blue) and the CK18 biomarker (green) and (b) segmented epithelial nuclei (blue), stroma nuclei (purple), and epithelial cytoplasm (green) [17].

This paper builds on previous work in IF biomarker quantification [25, 30]. Specifically, we analyzed expression of AR and Ki67 prostate biomarkers as proposed by Sapir et al. [25]. Quantification of a biomarker is achieved in two stages. First, a segmented tissue compartment is identified where the biomarker is expressed. Then, the signal is separated from the background within the compartment via intensity thresholding. Following the definition of epithelial and stromal nuclei, as well as epithelial cytoplasm, background autofluorescence and nonspecific binding effects are filtered out. An interactive model based thresholding technique is used to classify whether each of the nuclei is positive for a particular biomarker. The expression of each biomarker can then be quantized and normalized (epithelial signal normalized by stromal expression).  Figure 3 illustrates a multiplexed IF image and segmented epithelial versus stromal nuclei due to DAPI and CK18 markers. Features representing the relative rise of the biomarker in the epithelial disease specific compartments were recognized to be prognostic since they characterize the dynamic range of biomarker expression in an image [16, 17, 25].

## 4. Results and Discussion

### 4.1. Experiments with Clinical Characteristics

We applied the proposed transduction framework to build post-RP prognostic models using PSAR outcomes to predict the subsequent more advanced disease endpoint of CF. We analyzed three prostate cancer datasets of patients who had undergone radical prostatectomy. Dataset 1 [1] consisted of 262 patients with 8 clinical features, 37 of whom experienced PSAR (14% event rate). Dataset 2 [1] from a second institution consisted of 342 patients, 58 of whom experienced PSAR (17% event rate). Dataset 3 [1, 2] consisted of 340 new patients also from the second institution. Dataset 3 was unique because both the early PSAR endpoint and the later CF endpoint were available for all the patients. 43 patients experienced PSAR (13% event rate) and 12 experienced CF (3.5% event rate). Both Datasets 2 and 3 had 9 clinical features. The goal was to assess in Dataset 3 PSAR models built with Datasets 1 and/or 2.

We layered the transduction framework on top of both SVRc and the Cox Model and compared the performance with and without the transductive semi-supervised regression. We performed two rounds of experiments. In Table 1, we present the first where PSAR models were built with Dataset 1 and validated for both PSAR and CF with Dataset 3. In Table 2 we present the second round where PSAR models were built with Dataset 2 and validated for both PSAR and CF with Dataset 3. In developing medical prognostics, it is necessary to maintain separate training and validation sets (rather than combined cross-validation type approaches) due to FDA regulatory requirements for independent testing and validation. As noted, in all experiments the performance metrics were assessed according to the original times; no transduced targets were used in the accuracy assessments.

These prostate cancer experimental results appear to confirm the value of transductive semi-supervised regression for predicting late stage disease endpoints from earlier indications. For data from multiple institutions, existing survival analysis methods manifest an increase in empirical predictive accuracy when the transduction framework is layered on top. In all the experiments, whether we consider SVRc or the Cox model, in training and both validations, the transduction framework improves performance as measured by the defined Criterion in (11). While independent components of the criterion do vary, the algorithm was designed to optimize the overall criterion, and in this sense it has performed outstandingly.

Not only is the accuracy for PSAR improved, but, more importantly, CF is better predicted from the PSAR endpoint. In Table 2 there is a significant improvement in validation specificity due to the transduction approach. This makes sense because all the CF patients experienced PSAR and the PSAR assessment of high risk captures them, but it probably also has a high number of false positives since PSAR is a noisier endpoint and not all patients with PSAR experience CF. The accuracy of predicting CF is higher since CF is a more concrete and relevant endpoint.

Intuitively, the results when training on Dataset 2 and validating on Dataset 3, captured in Table 2, are better since both came from the same institution.

### 4.2. Deeper Dive on Features Driving Improvement

An interesting question to pose is whether there are differences in the features driving the improved prediction of validation performance for both SVRc and the Cox Model in the semi-supervised framework. We investigated the weights of all the clinical features in the models. It is difficult to compare the weights of feature across models; the magnitude of the weight only makes sense within the context of a particular model. Hence, we normalized the weights in each model accordingly by the highest weighted feature, thereby enabling a relative comparison of how important a particular feature is in a model.

One interesting observation to note is that, for both models with SVRc and the Cox Model, the dominant prostatectomy Gleason grade [1, 2, 21, 22] has a much higher relative weight in the transduction framework than in the models without the transduction framework. The implication being that this feature particularly is perhaps leading to an improved prediction. This is a noteworthy observation, since the role of the dominant prostatectomy Gleason for predicting CF is very interesting to urologists and oncologists. The status of seminal vesicle invasion also exhibits similar behaviour. There may be something meaningful in the interaction of these characteristics. This study was not designed to fully explore this insight, but it is worth considering in future work.

### 4.3. Expansion with Multimodal Imaging Characteristics

Since Datasets 2 and 3 were both from the same institution, IF and H&E images had been captured after all samples were similarly processed under an identical protocol. IF characteristics for Androgen Receptor (AR) were quantitated and H&E properties were extracted through automated image analysis. Models were again constructed for PSAR in Dataset 2 and validated for both PSAR and CF endpoints in Dataset 3. These results are presented in Table 4.

These results manifest that as new feature modalities are added to prostate cancer prediction models, the transduction framework continues to improve prediction performance. The overall performance criterion with transduction continues to outperform the nontransduction results for both SVRc and the Cox mode. Furthermore, the new feature domains of IF and H&E features in Table 4 improve results over their clinical only counterparts in Table 2. The only exception is the validation for PSAR results in the Cox model with transduction result.

Table 5 captures the original and normalized weights of these models. When comparing with the clinical only results in Table 3, it is evident that the new imaging modality features are now the most important ones in the models; they have some of the highest weights. This is particularly true for the SVRc results. Interestingly also, the dominant prostatectomy Gleason grade and seminal vesicle invasion status features are now no longer consistently doing better with transduction. In contrast, the PSA feature is now doing better with transduction for both SVRc and the Cox model. This may be due to the interaction of the PSA feature with some of the features, particularly the quantitative AR feature as PSA is a downstream marker of AR activity. Again, the study was not designed to deeply elucidate these connections, but in light of existing literature they are noteworthy and could be examined further. Overall, the results suggest meaningful improvements in performance with the transduction approach as multimodal characteristics are fused together to predict advanced prostate cancer from early PSAR.

### 4.4. Experiments with Multimodal Characteristics from Biopsy Data

The results presented thus far in Tables 1 through 5 represent models being built on features after RP. They represent clinical information known after surgical removal of a prostate, such as margin status, seminal vesicle invasion, and extracapsular extension status. Furthermore, imaging characteristics are extracted from tissue where areas of tumor have been definitively identified in the prostate specimen. Consequently, these models can be very accurate due to the relative wealth and robustness of disease specific information available.

Earlier in the disease progression timeline, newly diagnosed patients with a positive prostate biopsy and their physicians face a variety of potential treatment options including surgery, radiation therapy, active surveillance, and more. Which option is best for the individual patient is not always clear, and there have been a number of assays developed to analyze a patient's tumor specimen and provide a more personalized assessment of cancer severity and risk [3, 23]. Some of these assays employ image analysis algorithms to extract morphometric and biomolecular characteristics from the tumor specimen as features in predictive models for risk assessment. A practical challenge however is that there is often not enough tumor present in the biopsy specimen for analysis. Even if sufficient tumor is present, the amount of cancerous material may affect the accuracy of the predictive models [16].

For such patients, prognostic information after definitive therapy such as radical prostatectomy is still very useful in designing a treatment regimen. For instance, patients with poor predicted prognosis after RP would not be ideal candidates for active surveillance. Contrastingly, a patient with a high PSA and Gleason grade but good post-RP prognosis may be cured by a RP and is a likely candidate for surgery. A patient with low PSA and Gleason grade and still a good prognosis after RP may not need to undergo invasive surgery with all its complications and could be served by less severe interventions.

Consequently, the availability of post-RP prognosis based on clinical characteristics available at the time of biopsy, and analysis of the biopsy tumor specimen, is a valuable resource. These models are less accurate than the RP based models since less clinical information is known and the variability of the tumor is large, but they are still important. Similar challenges exist where post-RP PSAR endpoints are more readily available and can help predict more advanced CF.

Dataset 4 [3] consists of 1027 patients with 3 clinical features available at the time of biopsy and 9 multimodal characteristics extracted from quantitative image analysis of the variable tumor in biopsy samples. These include measurements of AR and Ki67 biomarker expression, as well as H&E image analysis. Both the earlier PSAR endpoint and later CF endpoints were available for all patients. The data was split into 686 training and 341 validation patients. Models were built for the PSAR endpoint and validated for both PSAR and CF. Results are presented in Table 6.

With less information available at the time of biopsy, these models are less accurate overall. However, the same pattern in performance can be observed. The transduction framework is improving prediction for both PSAR and CF in validation, with SVRc and the Cox model. It is very interesting to note that, for CF, there continues to be a noticeable improvement in specificity rather than sensitivity. As presented in Table 7, the Gleason sum appears to consistently be more important in the transduction models.

## 5. Conclusions

This paper presents strong evidence supporting the value of a novel transductive semi-supervised regression framework for the challenging problem of predicting advanced prostate cancer from earlier disease endpoints. In multiple experiments from different datasets of both prostate biopsy and prostatectomy cohorts, the transductive framework yields improvements in the prognostic performance of prostate cancer prediction models. Prostate cancer prediction is rapidly integrating different information domains such as clinical, protein expression, and imaging characteristics together into multivariate analyses. Overall, the results suggest meaningful improvements in performance with the transduction approach as multimodal characteristics are fused together to predict advanced prostate cancer from early PSAR. This work presents one of the first innovative applications of this recently developed transduction technique for predicting subsequent endpoints from earlier ones and may be useful in other diseases as well, not just prostate cancer. In the future we plan to evaluate additional survival analysis algorithms and to explore other performance criteria.

## Figures and Tables

**Table 1:** Results of training on Dataset 1 and validating on Dataset 3.

	***SVRc***	***SVRc with Transduction***	***Cox Model***	***Cox Model with Transduction***
	**PSAR Training Performance**			

CI	0.79	0.81	0.80	0.80

Sensitivity	0.77	0.87	0.80	0.70

Specificity	0.76	0.72	0.73	0.85

**Criterion**	**1.38**	**1.44**	**1.38**	**1.40**

	**PSAR Validation Performance**			

CI	0.74	0.76	0.77	0.80

Sensitivity	0.79	0.90	0.79	0.69

Specificity	0.62	0.58	0.59	0.75

**Criterion**	**1.23**	**1.28**	**1.24**	**1.32**

	**CF Validation Performance**			

CI	0.76	0.78	0.79	0.79

Sensitivity	0.83	1.00	1.00	1.00

Specificity	0.58	0.53	0.57	0.72

**Criterion**	**1.24**	**1.31**	**1.36**	**1.51**

**Table 2:** Results of training on Dataset 2 and validating on Dataset 3.

	***SVRc***	***SVRc with Transduction***	***Cox Model***	***Cox Model with Transduction***
	**PSAR Training Performance**			

CI	0.78	0.79	0.80	0.81

Sensitivity	0.77	0.68	0.82	0.77

Specificity	0.73	0.83	0.71	0.75

**Criterion**	**1.34**	**1.35**	**1.38**	**1.39**

	**PSAR Validation Performance**			

CI	0.80	0.81	0.82	0.82

Sensitivity	0.74	0.69	0.79	0.79

Specificity	0.72	0.83	0.72	0.82

**Criterion**	**1.33**	**1.38**	**1.39**	**1.47**

	**CF Validation Performance**			

CI	0.88	0.88	0.88	0.88

Sensitivity	1.00	1.00	1.00	1.00

Specificity	0.68	0.78	0.68	0.75

**Criterion**	**1.56**	**1.66**	**1.56**	**1.63**

**Table 3:** Weights of features in models.

	Cox Model		Cox Model with Transduction		SVRc		SVRc with Transduction	
Feature	Original Weight	Normalized Weight	Original Weight	Normalized Weight	Original Weight	Normalized Weight	Original Weight	Normalized Weight

Clinical Stage	0.314	0.217	0.355	0.316	-2.939	-0.12	-4.496	-0.197

PSA	0.743	0.513	0.818	0.728	-10.329	-0.423	-8.397	-0.368

Dominant Biopsy Gleason Grade	-0.198	-0.137	0.134	0.119	-3.909	-0.16	-8.193	-0.359

Biopsy Gleason Sum	1.448	1	1.124	1	-24.394	-1	-22.826	-1

Dominant Prostatectomy Gleason Grade	0.872	0.602	1.002	0.891	-4.363	-0.179	-6.433	-0.282

Prostatectomy Gleason Sum	0.073	0.05	-0.139	-0.123	-11.852	-0.486	-8.784	-0.385

Seminal Vesicle Invasion	0.747	0.516	0.796	0.708	-23.926	-0.981	-25.963	-1.137

Surgical Margin Status	0.306	0.212	0.261	0.232	-2.778	-0.114	-3.964	-0.174

Extra Capsular Extension Status	0.198	0.137	0.161	0.143	-4.259	-0.175	-3.499	-0.153

**Table 4:** Results Training on Dataset 2 and Validating on Dataset 3 with Multimodal Characteristics.

	***SVRc***	***SVRc with Transduction***	***Cox Model***	***Cox Model with Transduction***
	**PSAR Training Performance**			

CI	0.80	0.81	0.81	0.82

Sensitivity	0.79	0.82	0.79	0.79

Specificity	0.75	0.78	0.80	0.82

**Criterion**	**1.39**	**1.45**	**1.44**	**1.47**

	**PSAR Validation Performance**			

CI	0.79	0.80	0.80	0.82

Sensitivity	0.79	0.83	0.78	0.78

Specificity	0.73	0.77	0.78	0.81

**Criterion**	**1.37**	**1.44**	**1.41**	**1.45**

	**CF Validation Performance**			

CI	0.9	0.9	0.92	0.93

Sensitivity	1	1	1	1

Specificity	0.70	0.81	0.73	0.76

**Criterion**	**1.60**	**1.71**	**1.65**	**1.69**

**Table 5:** Weights of features in models with multimodal characteristics.

	Cox Model		Cox Model with Transduction		SVRc		SVRc with Transduction	
Feature	Original Weight	Normalized Weight	Original Weight	Normalized Weight	Original Weight	Normalized Weight	Original Weight	Normalized Weight

Clinical Stage	0.475	0.408	0.535	0.444	-3.083	-0.174	-6.658	-0.320

PSA	0.700	0.601	0.893	0.741	-9.763	-0.552	-15.587	-0.749

Dominant Biopsy Gleason Grade	-0.094	-0.081	0.039	0.032	-4.867	-0.275	-6.679	-0.321

Biopsy Gleason Sum	1.164	1.000	1.205	1.000	-16.801	-0.949	-20.076	-0.965

Dominant Prostatectomy Gleason Grade	0.455	0.391	0.643	0.534	-7.005	-0.396	-7.460	-0.358

Prostatectomy Gleason Sum	0.260	0.223	0.181	0.150	-14.282	-0.807	-7.011	-0.337

Seminal Vesicle Invasion	0.842	0.723	0.846	0.703	-20.542	-1.161	-24.166	-1.161

Surgical Margin Status	0.371	0.319	0.414	0.344	-4.788	-0.271	-6.757	-0.325

Extra Capsular Extension Status	0.802	0.689	0.892	0.740	-6.217	-0.351	-6.485	-0.312

AR+ Area	-1.099	-0.944	-1.026	-0.852	15.277	0.863	17.347	0.833

Cytoplasm Texture	-1.195	-1.026	-0.931	-0.773	17.699	1.000	20.814	1.000

Luminal Area	-0.467	-0.401	-0.424	-0.352	10.264	0.580	12.092	0.581

Glandular Size	0.475	0.408	0.535	0.444	-3.083	-0.174	-6.658	-0.320

**Table 6:** Results of training on Dataset 4 with multimodal characteristics from biopsy samples.

	***SVRc***	***SVRc with Transduction***	***Cox Model***	***Cox Model with Transduction***
	**PSAR Training Performance**			

CI	0.69	0.69	0.69	0.69

Sensitivity	0.70	0.63	0.62	0.61

Specificity	0.61	0.73	0.71	0.76

**Criterion**	**1.12**	**1.15**	**1.13**	**1.15**

	**PSAR Validation Performance**			

CI	0.69	0.70	0.69	0.69

Sensitivity	0.70	0.61	0.61	0.59

Specificity	0.60	0.73	0.71	0.74

**Criterion**	**1.11**	**1.15**	**1.12**	**1.13**

	**CF Validation Performance**			

CI	0.60	0.60	0.6	0.61

Sensitivity	0.67	0.67	0.57	0.57

Specificity	0.54	0.62	0.63	0.66

**Criterion**	**0.96**	**1.02**	**0.96**	**0.99**

**Table 7:** Weights of features in models with multimodal characteristics from biopsy data.

	Cox Model		Cox Model with Transduction		SVRc		SVRc with Transduction	
Feature	Original Weight	Normalized Weight	Original Weight	Normalized Weight	Original Weight	Normalized Weight	Original Weight	Normalized Weight

PSA	1.715	1.000	1.720	1.000	-60.799	-5.430	-62.229	-6.859

Dominant Biopsy Gleason Grade	0.565	0.329	0.467	0.272	-21.025	-1.878	-23.007	-2.536

Biopsy Gleason Sum	0.476	0.278	0.586	0.341	-22.943	-2.049	-22.912	-2.525

AR Expression	0.251	0.146	0.185	0.108	-18.932	-1.691	-19.903	-2.194

Nuclear Morphology	-0.228	-0.133	-0.263	-0.153	-0.866	-0.077	-0.001	0.0001

Ki67+ Area	0.885	0.516	0.793	0.461	-28.498	-2.545	-28.366	-3.127

Luminal Area	-0.171	-0.100	-0.159	-0.093	11.197	1.000	9.073	1.000

Epithelial Cells Infiltration	-0.127	-0.074	-0.161	-0.094	-2.226	-0.199	-3.040	-0.335

Glandular Size	0.088	0.052	0.019	0.011	-9.765	-0.872	-8.777	-0.967

## Data Availability

Data for the study is available by contacting the author. Restrictions on use may exist and a usage agreement may need to be signed.

## References

[1] Cordon-Cardo C., Kotsianti A., Verbel D. A. (2007). Improved prediction of prostate cancer recurrence through systems pathology. *The Journal of Clinical Investigation*.

[2] Donovan M. J., Hamann S., Clayton M. (2008). Systems pathology approach for the prediction of prostate cancer progression after radical prostatectomy. *Journal of Clinical Oncology*.

[3] Donovan M., Khan F. M., Fernandez G. (2009). Personalized Prediction of Tumor Response and Cancer Progression from the Prostate Needle Biopsy. *The Journal of Urology*.

[4] Khan F. M., Liu Q. (2011). Medical Survival Analysis Through Transduction of Semi-Supervised Regression Targets. *International Journal of Knowledge Discovery in Bioinformatics*.

[5] Khan F. M., Bayer-Zubek V. Support vector regression for censored data (SVRc): a novel tool for survival analysis.

[6] Shiao H.-T., Cherkassky V. Learning using privileged information (LUPI) for modeling survival data.

[7] Burke H. B., Goodman P. H., Rosen D. B. (1997). Artificial neural networks improve the accuracy of cancer survival prediction. *Cancer*.

[8] Snow P. B., Smith D. S., Catalona W. J. (1994). Artificial neural networks in the diagnosis and prognosis of prostate cancer: A pilot study. *The Journal of Urology*.

[9] Harrell F. E. (2001). *Regression Modeling Strategies with Applications to Linear Models, Logistic Regression, and Survival Analysis*.

[10] Therneau T. M., Grambsch P. M. (2000). *Modeling Survival Data: Extending the Cox Model*.

[11] Chapelle O., Sindhwani V., Keerthi S. S. (2008). Optimization techniques for semi-supervised support vector machines. *Journal of Machine Learning Research*.

[12] Chen Y., Wang G., Dong S. (2003). Learning with progressive transductive support vector machine. *Pattern Recognition Letters*.

[13] Seeger M., Chappelle O., Scholkopf B., Zien A. (2006). A taxonomy of semi-supervised methods. *Semi-Supervised Learning*.

[14] Cortes C., Mohri M. On transductive regression.

[15] Khan F. M., Liu Q. Transduction of semi-supervised regression targets in survival analysis for medical prognosis.

[16] Khan F. M., Kulikowski C. A. Impact of prostate biopsy tumor amount on imaging based prognostics employing transductive semi-supervised regression.

[17] Khan F. M., Kulikowski C. A. The role of imaging based prostate biopsy morphology in a data fusion paradigm for transducing prognostic predictions.

[18] Papadimitriou C. H. (1994). *Computational Complexity*.

[19] Sedgewick R., Wayne K. (2011). *Algorithms*.

[20] Raykar V. C., Steck H., Krishnapuram B., Dehing-Oberije C., Lambin P. On ranking in survival analysis: Bounds on the concordance index.

[21] Epstein J. (1995). *Prostate Biopsy Interpretation*.

[22] Gleason D. (1977). *The Veteran’s Administration Cooperative Urologic Group: Histologic gRading And Clinical Staging of Prostatic Carcinoma,” Urologic Pathology: The Prostate*.

[23] Blume-Jensen P., Berman D. M., Rimm D. L. (2015). Development and Clinical Validation of an In Situ Biopsy-Based Multimarker Assay for Risk Stratification in Prostate Cancer. *Clinical Cancer Research*.

[24] Fogarasi S., Khan F. M., Pang H.-Y. H., Mesa-Tejada R., Donovan M. J., Fernandez G. Glandular Object Based Tumor Morphometry in H&E Biopsy Samples for Prostate Cancer Prognosis.

[25] Sapir M., Khan F. M., Vengrenyuk Y. Improved automated localization and quantification of protein multiplexes via multispectral fluorescence imaging in heterogenous biopsy samples.

[26] Tabesh A., Karssemeijer N., Giger M. L. Robust tumor morphometry in multispectral fluorescence microscopy.

[27] Doyle S., Hwang M., Shah K., Madabhushi A., Feldman M., Tomaszeweski J. Automated grading of prostate cancer using architectural and textural image features.

[28] Leo P., Lee G. Evaluating stability of histomorphometric features across scanner and staining variations: Predicting biochemical recurrence from prostate cancer whole slide images.

[29] Lopez C. M., Agaian S., Sanchez I. Exploration of efficacy of gland morphology and architectural features in prostate cancer gleason grading.

[30] Teverovskiy M., Vengrenyuk Y., Tabesh A. Automated localization and quantification of protein multiplexes via multispectral fluorescence imaging.

[31] Khan F. M., Kulikowski C. A. Predicting Advanced Prostate Cancer Endpoints from Early Indications via Transductive Semi-Supervised Regression.

